# Expanding the Landscape of Amino Acid-Rich Antimicrobial Peptides: Definition, Deployment in Nature, Implications for Peptide Design and Therapeutic Potential

**DOI:** 10.3390/ijms232112874

**Published:** 2022-10-25

**Authors:** Aaron P. Decker, Abraham F. Mechesso, Guangshun Wang

**Affiliations:** Department of Pathology and Microbiology, College of Medicine, University of Nebraska Medical Center, 985900 Nebraska Medical Center, Omaha, NE 68198-5900, USA

**Keywords:** antibiotic resistance, antimicrobial peptides, antimicrobial peptide database, bioinformatics, peptide design, peptide antibiotics

## Abstract

Unlike the α-helical and β-sheet antimicrobial peptides (AMPs), our knowledge on amino acid-rich AMPs is limited. This article conducts a systematic study of rich AMPs (>25%) from different life kingdoms based on the Antimicrobial Peptide Database (APD) using the program R. Of 3425 peptides, 724 rich AMPs were identified. Rich AMPs are more common in animals and bacteria than in plants. In different animal classes, a unique set of rich AMPs is deployed. While histidine, proline, and arginine-rich AMPs are abundant in mammals, alanine, glycine, and leucine-rich AMPs are common in amphibians. Ten amino acids (Ala, Cys, Gly, His, Ile, Lys, Leu, Pro, Arg, and Val) are frequently observed in rich AMPs, seven (Asp, Glu, Phe, Ser, Thr, Trp, and Tyr) are occasionally observed, and three (Met, Asn, and Gln) were not yet found. Leucine is much more frequent in forming rich AMPs than either valine or isoleucine. To date, no natural AMPs are simultaneously rich in leucine and lysine, while proline, tryptophan, and cysteine-rich peptides can simultaneously be rich in arginine. These findings can be utilized to guide peptide design. Since multiple candidates are potent against antibiotic-resistant bacteria, rich AMPs stand out as promising future antibiotics.

## 1. Introduction

Host defense antimicrobial peptides (AMPs) are universally deployed in nature. As key elements of innate immunity, they can be constitutively expressed or induced by invading pathogens to protect plants and animals from infection. They may also regulate the immune response by interacting with cell receptors [1,2,3,4,5]. According to the antimicrobial peptide database (APD) [6,7,8], AMPs have been discovered from the six life kingdoms: bacteria, archaea, protists, fungi, plants, and animals [8]. These peptides are usually small with 11–50 amino acids. Although well-known for cationicity, the net charge of AMPs can range from −12 to +30 with the maximum at +3. The majority of AMPs have hydrophobic contents between 31% and 60%, but some can be entirely hydrophobic or hydrophilic. An improved understanding of these peptides is essential for developing them into a new generation of antibiotics to combat drug-resistant pathogens.

Initially, AMPs were separated into three classes: α-helix, β-sheet, and amino acid rich [9]. The helical family is best known for its amphipathic nature. In the APD [8], the α-helical scaffold has been found in all the six kingdoms [10,11,12,13]. Such an amphipathic structure can initiate bacterial targeting via electrostatic attractions between the cationic amino acids and anionic surfaces of bacteria followed by membrane anchoring through hydrophobic interactions. Multidimensional nuclear magnetic resonance (NMR) spectroscopy has proved the existence of such an amphipathic structure in membrane-mimicking models and provided unambiguous evidence for interactions between peptide and phosphatidylglycerols (PGs) [14]. Depending on the peptide nature and concentration, membrane active peptides may act by causing a series of membrane phase changes [7], including lipid domain formation, pore formation, and membrane disruption into smaller pieces [15,16,17]. AMPs in the second class have a β-sheet structure. A typical example is α-defensins isolated from humans [18]. The crystallographic analysis reveals three strands in the β-sheet structure of α-defensin, which is further stabilized by three disulfide bonds [19]. Moreover, the β-sheet of α-defensin can pack with each other in the crystal. The formation of nanofibers in the case of human HD6 is proposed to tangle bacteria [20]. Interestingly, HD6 becomes bactericidal only after reduction [21]. Depending on the microorganism, defensins could kill bacteria by targeting membranes or cell walls [22].

Amino acid-rich antimicrobial peptides (i.e., rich AMPs) from the third class were thought to be able to form neither an α-helix nor a β-sheet structure. Because of sequence uniqueness and the repetition of the same amino acids, these peptides possess special properties. The classic rich AMP families are His-rich, Gly-rich, Pro-rich, Trp-rich, and Arg-rich [23,24,25,26]. Although occasionally found in nature, Trp-rich peptides are widely investigated in laboratories [27,28,29]. One of the important reasons is that Trp-rich AMPs can be made at a relatively short length to reduce the cost of peptide production. These classic rich AMPs were first detected by human eyes due to the presence of numerous identical amino acids in the sequence. However, a general and quantitative criterium for rich AMPs was lacking. In 2010, we made an attempt to define rich AMPs in the APD where the content of one amino acid was greater than 25% [7,30]. Recently, Australian researchers made the same definition for Pro-rich AMPs [31]. To our knowledge, an unbiased analysis of rich AMPs for all 20 amino acids has not been pursued.

This article reports the identification of all rich AMPs in the APD. We chose the APD for this analysis because it is an original database dedicated to natural antimicrobial peptides from all life kingdoms. During the past 19 years, the peptide number has increased from the original 525 to the current 3243, facilitating this study [6,7,8]. In this article, we first provide a definition for rich AMPs and discuss exceptions. We then identify and describe all the amino acids with examples of rich AMPs in different life kingdoms. In addition, we have mapped the distribution patterns of rich AMPs in the animal kingdom. Our work significantly expands the scope of rich AMPs beyond a few classic families. Our study also shines light on peptide design based on Nature’s wisdom. Finally, we discuss rich AMPs that will likely become future antibiotics on the basis of numerous rich AMP examples with demonstrated in vivo efficacy in murine models [32,33,34,35,36,37].

## 2. Results

### 2.1. Rich Antimicrobial Peptides in the Six Life Kingdoms

**Figure 1:**
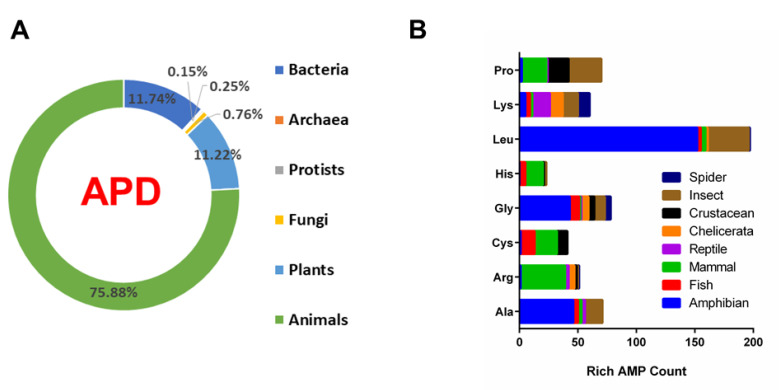
(**A**) Counts of antimicrobial peptides from the six life kingdoms. There are 385 peptides from bacteria (12%), 5 from archaea, 8 from protists, 25 from fungi, 368 from plants (11%), and 2489 from animals (76%). Peptides from archaea, protists, and fungi are limited (1%). (**B**) Distribution of amino acid rich antimicrobial peptides in a variety of animal classes. Data were obtained from the antimicrobial peptide database (https://aps.unmc.edu) on 3 June 2022.

The amino acid compositions (in percentage) of 3425 antimicrobial peptides registered in the APD were calculated and are available upon request. The sequence was annotated as amino acid rich in the APD if the content of one amino acid is above 25%. In total, we identified 724 rich AMPs in the current database, accounting for 21%. Because AMPs from archaea, protists, and fungi are limited in the current APD (1%), we focused our attention on the rich AMPs from bacteria, plants, and animals with 385, 368, and 2489 peptides, respectively, the sum of which accounts for 99% of natural AMPs in the database (Figure 1). We identified 63 rich AMPs from bacteria (16%), 12 from plants (3%), and 598 from animals (24%). Hence, animals possess the highest percentages of rich AMPs followed by bacteria. Of note, the percentage of rich AMPs in plants is remarkably low.

Based on the total number of rich AMP for each amino acid from bacteria, plants and animals, we classified them into three groups: frequent (>10 counts), occasional (<10 counts), and never (no count in Table 1). The ten frequently observed rich AMP groups include Ala-rich, Cys-rich, Gly-rich, His-rich, Ile-rich, Lys-rich, Leu-rich, Pro-rich, Arg-rich, and Val-rich. This list includes those classic rich AMPs. In this list, Leu-rich AMPs, with 212 peptides, are most abundant, and Val-rich peptides are the least abundant with only 14 counts. The occasionally observed groups cover seven amino acids: Asp-rich, Glu-rich (acidic), Ser-rich, Thr-rich (polar), Phe-rich, Trp-rich and Tyr-rich (aromatic). Finally, we did not find any members for Met-rich, Asn-rich, and Gln-rich AMPs in the APD.

In bacteria, only Gly-rich, Leu-rich, and Val-rich AMPs had over 10 counts. It is interesting to note that most of the Val-rich AMPs are made in bacteria. In addition, the three Thr-rich AMPs in the APD are all bacterial peptides. They have not been found in plants or animals. In plants, 14 amino acids showed no examples of rich AMPs (Table 1). The remaining six amino acids (Gly, His, Lys, Leu, Pro, and Arg) had less than five counts. In animals, we found over 10 counts for Ala, Arg, Cys, Gly, His, Ile, Leu, Lys, and Pro. It is useful to mention the averaged rich amino acids in Table 1. Except for His-rich (25.1%) and Cys-rich AMPs (23.1%), other rich AMPs had average values around 30%. Only Gly-rich and Asp-rich AMPs showed higher averages.

### 2.2. Distribution of Rich Antimicrobial Peptides in Animals

We then further analyzed the distribution of the nine families of frequently observed rich AMPs in 10 animal groups (Table 2). This distribution for a select set of rich AMPs is summarized in Figure 1B. From 1148 amphibian AMPs [10,38,39], we found 46 Ala-rich, 42 Gly-rich, and 152 Leu-rich AMPs. This is an interesting discovery, since these amino acids are all frequently occurring in the amino acid signature plot [30]. Surprisingly, there were only five Lys-rich AMPs. It is not clear why Lys-rich is the least favored in amphibians. In 352 mammalian AMPs, we found 19 Cys-rich, 15 His-rich, 22 Pro-rich, and 38 Arg-rich peptides. While most of the 19 Cys-rich AMPs are θ-defensins (smallest defensins in nature), His-rich AMPs are limited to histatins [26]. Among the 22 Pro-rich peptides from mammals, 14 are cathelicidins. Likewise, 18 out of 38 Arg-rich AMPs are cathelicidins [23,24,25,26,27,28,29]. Moreover, Pro-rich AMPs are frequently accompanied by arginine. When both Arg-rich and Pro-rich were included in the database search, we obtained 10 rich AMPs from mammals. Interestingly, Arg-rich AMPs are only abundant in mammals (>10 counts). The APD collected 339 insect AMPs. Five amino acids (Ala, Gly, Lys, Leu, and Pro) gave a good number of rich AMPs (9–29 counts in Table 2). Only amphibians shared Ala-, Gly-, and Leu-rich AMPs with insects. Different from mammals, the counts of Lys-rich AMPs in insects, reptiles, spiders, and chelicerata are all above 10. It appears that rich AMPs are deployed differently in various animal groups (Table 2). This could be a unique aspect for each host in fending off invading pathogens. 

### 2.3. Length-Dependent Probing of Rich Antimicrobial Peptides with Amino Acid Homopolymers

**Figure 2:**
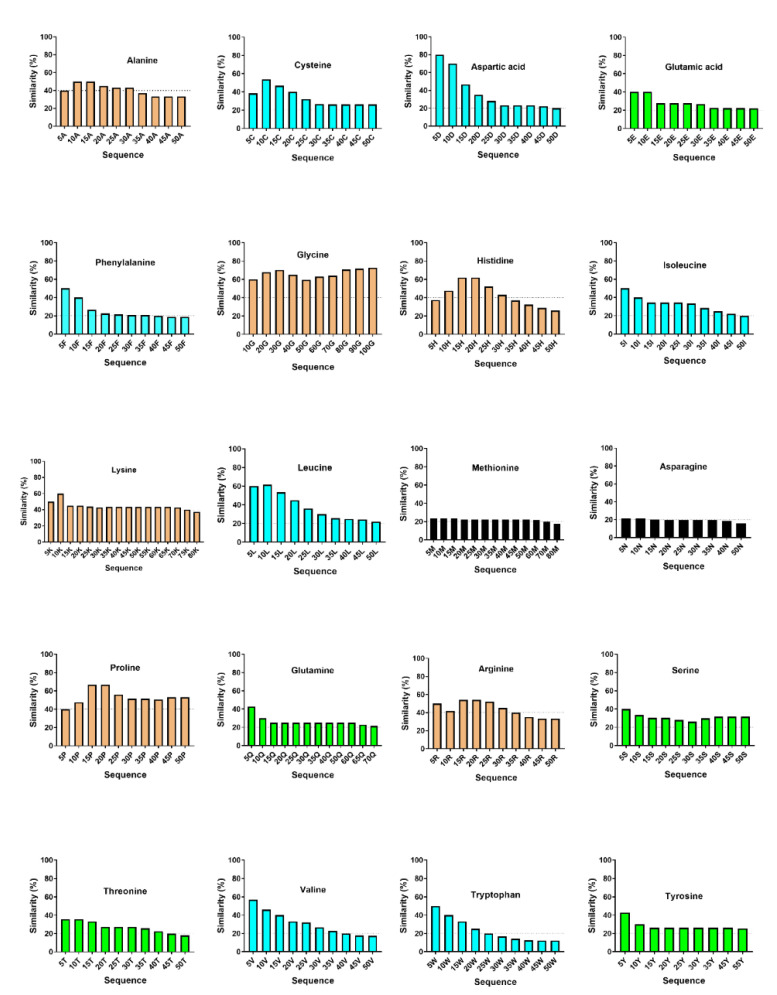
Homopolymer probing of rich antimicrobial peptides. The molecular probe for each of the standard 20 amino acids consists of a string of 5, 10, 15, 20, … amino acids at a step size of 5, ranging from 5 to 50. The probe was utilized to identify the most similar antimicrobial peptides in the APD prediction interface. The plot is a function of peptide similarity with the probe length. The plots are classified into four groups: High and wide similarity (gold), clear similarity decrease with the probe length (cyan), low similarity (green), and no similarity (black).

How AMPs appeared in nature is not known. One possibility is that they started from amino acid homopolymers followed by site-directed mutations. Although this hypothesis is challenging to test, it is possible to search homopolymer-like AMPs in the APD that are most similar to the probe molecules at different lengths (step size 5). The results are plotted in Figure 2 for each amino acid. Six amino acids showed good similarity (around 40%) in a broad range of up to 50 amino acids. They are small alanine, glycine, turn-forming proline, basic histidine, lysine, and arginine (gold in Figure 2), indicating the flexibility of these amino acids in forming rich AMPs at various lengths. As the second group, cysteine, aspartic acid, phenylalanine, isoleucine, leucine, valine, and tryptophan-rich AMPs (cyan) also have a high content at short lengths in Figure 2, but they rapidly decayed to the baseline level at about 30 amino acids. Some amino acids showed high similarity only when the probe length was very short. Then, the similarity decayed to ≈20%. Included in the third group were glutamic acid, glutamine, serine, threonine, and tyrosine (green). Of particular interest are the flat plots for methionine and asparagine (Figure 2 black) at about 20%. Both showed poor similarity in the entire peptide range we probed. Glutamine could have given the same flat plot if we had not included a short and incomplete sequence in this analysis. These results are consistent with the fact that Met-, Asn-, and Gln-rich AMPs have not yet been discovered (Table 1). In addition, our molecular probing using homopolymers provided insight into the length-dependent formation for rich AMPs. Indeed, our database search revealed that the peptide lengths of 84 Ala-rich AMPs range from 10 to 69. Lys-rich AMPs are broad in length as well. The shortest peptide has only nine amino acids, while the longest one is 69 residues long. Arg-rich AMPs span from eight to 94 amino acids. Pro-rich AMPs range from nine to 94 amino acids (even longer sequences contain two domains). There are 106 Gly-rich AMPs in the APD with peptide lengths from nine to 155. Ile-, Leu-, Trp-, and Val-rich AMPs are limited to about 30–35 amino acids. Moreover, some rich AMPs are relatively short. Asp-rich AMPs have five to seven amino acids, whereas Phe-rich AMPs range from seven to 15 residues with three to four phenylalanine residues in the sequences. 

### 2.4. Amino Acid Distribution in Selected Rich Antimicrobial Peptides

**Figure 3:**
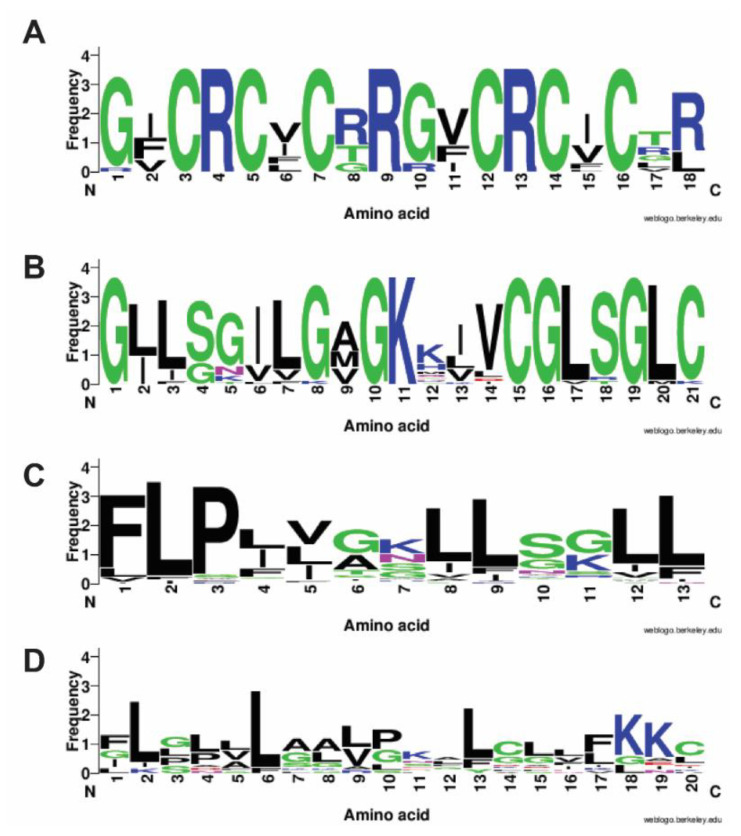
Logo plots of select rich antimicrobial peptides: (**A**) 19 Cys-rich θ-defensins with 18 amino acids; (**B**) 23 Gly-rich nigrocins with 21 amino acids; (**C**) 49 Leu-rich temporins with 13 amino acids; (**D**) 13 Leu-rich AMPs with 20 amino acids. The figure was made using the web logo at https://weblogo.berkeley.edu/logo.cgi accessed on 3 June 2022.

To provide insight into the amino acid use, we generated logo plots [40] for select families of rich AMPs. Figure 3A is the plot for 19 θ-defensins (three synthetic and 16 natural) with a fixed length of 18 amino acids. Cysteine fully occupies positions 3, 5, 7, 12, 14, and 16. In addition, arginine is also a fixed residue at positions 4, 9, and 13. This residue also has some preference for positions 8 and 18. The Gly-rich feature can be seen in Figure 3B for 23 amphibian nigrocins with 21 residues. Glycine dominates at positions 1, 8, 10, 16, and 19. Moreover, two cysteines occupy positions 15 and 21 at the C-terminus, forming the Rana box, which has a conserved sequence CGLXGXC. Figure 3C is a plot of 49 temporins at a constant length of 13 [4]. The plot reveals the dominance of leucine at positions 2, 8, 9, 12, and 13, while position 1 is frequently occupied by phenylalanine. In addition, a proline is evident at position 3. Since there are over 200 Leu-rich AMPs in the APD, we also made a plot for 13 Leu-rich peptides with 20 amino acids in the database (Figure 3D). Unlike the temporin family, these peptides are rather diverse. However, the preference of leucine at positions 2, 6, and 13 can be seen. It is also at the top at positions 4, 9, and 15. A lysine pair stands out at the C-terminus. These plots depict drastically different sequence patterns for rich AMP models designed by nature, probably to meet the requirement of eliminating a variety of pathogens rapidly.

### 2.5. Structure of Amino Acid-Rich Antimicrobial Peptides

**Figure 4:**
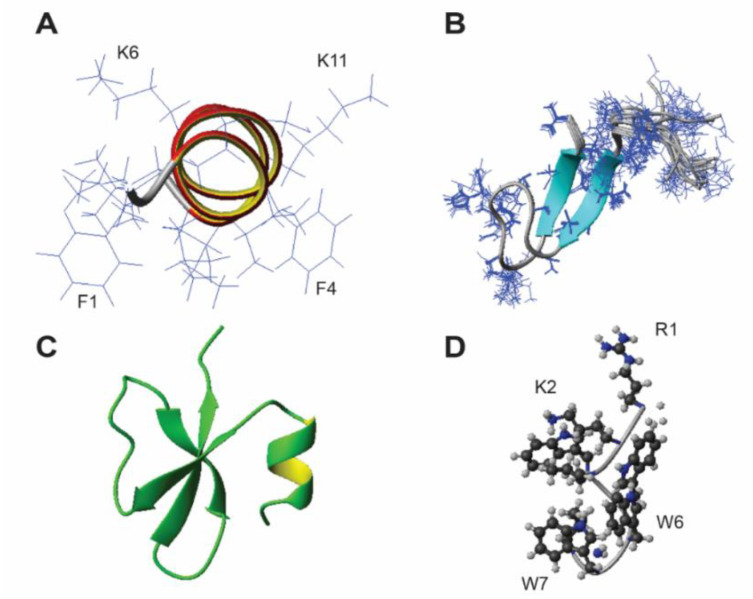
NMR structures of representative rich antimicrobial peptides. (**A**) Leu-rich amphibian temporin-1OLa in complex with deuterated sodium dodecylsulfate micelles (molar ratio 1:60) (Wang, G., coordinates not deposited); (**B**) Cys-rich human hepcidin (PDB ID: 1M4F) as a hormone to regulate iron use; (**C**) king penguin Arg-rich β-defensin spheniscin for food preservation during egg incubation (PDB ID: 1UT3); (**D**) Trp-rich WW295 bound to micelles, the parent peptide of verine designed based on the antimicrobial peptide database (PDB ID: 6NM3).

Based on the presence or absence of α-helix and β-sheet, the 3D structures of AMPs are classified into four self-consistent families: α, β, αβ, and non-αβ in a more recent classification scheme [6]. Figure 4 shows the four representative structures of rich AMPs. Table 3 documents the known structures in the APD for each amino acid-rich AMPs. Among these rich peptides, 12 types of amino acids (60%) have at least one known structure. The α-helix structure was by far the most prevalent for rich AMPs (Figure 4A). For Ala-rich, His-rich, Phe-rich, and Val-rich, only helical structures were found. The β-sheet structure was primarily found in Arg-rich and Cys-rich peptides. While cysteines can form disulfide bonds to stabilize the β-sheet structure (Figure 4B), arginines are critical for antimicrobial activities. Despite only one example in the APD, penguin β-defensin AvBD103b is rich in arginine and has a mixture of α and β structures (Figure 4C). Non-αβ structures were found in Arg-rich, Gly-rich, Pro-rich, and Trp-rich AMPs. Figure 4D shows a novel non-αβ spiral structure determined for a designed Trp-rich peptide [34]. Multiple extended structures were found for Trp-rich peptides as well [27], providing evidence for the original rich classification [9]. However, Trp-rich AMPs can also form helical structures. The parent peptide for Trp-rich horine is helical [34]. To date, no 3D structure has been determined for Asp-rich, Glu-rich, Ser-rich, Thr-rich, and Tyr-rich peptides (Table 3). Hence, rich AMPs can form α-helix (139 rich AMPs), β-sheet (24 examples), αβ-structure (1 peptide), and non-αβ structures (7 examples). These examples indicate that rich AMPs can adopt all kinds of structures; they are not limited to the classic “extended” structure, which is a special case of the non-αβ family. Our analysis implies that “richness” is a unique peptide property and can be used as an independent criterium for peptide classification, prediction and design. Based on the 25% definition, 21% AMPs in the APD are rich, and 79% are normal (not rich) AMPs.

### 2.6. Activity of Amino Acid Rich Antimicrobial Peptides

To map the activity spectrum, Table 4 lists rich AMP counts with antibacterial, antifungal, antiviral, and antiparasitic activities in the APD. Most of the peptides, especially Leu-, Gly, Arg-, Lys-, Ala-, and Pro-rich AMPs, exhibited a broad spectrum of activity against bacteria and fungi. In addition, some rich AMPs are antiviral (Arg- and Cys-rich) and antiparasitic (Leu-rich). It is useful to mention that rich AMPs may have selective antibacterial activity. As shown in Table 4, some Leu-rich AMPs (e.g., temporins) primarily kill Gram-positive pathogens [4], whereas Pro-rich AMPs are known to inhibit the protein synthesis of Gram-negative pathogens [31,32]. The preferential bacterial killing of these peptides can also be seen by searching a specific pathogenic species in the APD as well. We found 138 Leu-rich AMPs active against *Staphylococcus aureus* and 81 against *Escherichia coli*. Likewise, there were 39 Pro-rich peptides against *E. coli* and 15 against *S. aureus* in the current database. Pro-rich AMPs mainly target the 70S ribosomes to inhibit bacterial protein translation and interfere with protein synthesis [41,42]. Some Pro-rich AMPs such as arasin 1 exhibit a dual mode of action with both membrane disruption and probable intracellular target inhibition [43,44]. In addition, Pro-rich AMPs may regulate immune response [44,45,46]. Rich AMPs also have other biological functions, including anticancer, toxin neutralization, chemotactic, anti-inflammation, antioxidant, and wound healing. These peptide functions are annotated in the APD [8,47], and interested readers can search the database for a detailed list of AMPs with such properties.

## 3. Discussion

Amino acid richness is an important feature of natural antimicrobial peptides. In the literature, the meaning of the word “rich” is paper-dependent and not consistently defined. However, they all convey the same message that certain amino acids appear multiple times in the sequence. Usually, the word “rich” is limited to a set of classic peptides such as His-rich, Trp-rich, Gly-rich, and Arg-rich. Hence, our study has filled in this knowledge gap and expanded the rich AMPs from a few classic examples to 17 amino acids (Table 1). Only methionine, asparagine, and glutamine did not have any examples in the APD. The lack of Met-rich AMPs may be due to the readiness of oxidation of this residue, especially when it is located on the hydrophobic surface, causing a loss of antimicrobial activity as observed in vitro [48,49]. Of note, Met-rich regions can occur in proteins. For example, a Met-rich region is deployed in a membrane protein reductase by accelerating the reaction between serotonin and copper [50]. Likewise, Asn-rich and Gln-rich sequences have been discovered in numerous proteins. They are proposed to be prion domains of proteins involved in aggregation and amyloid formation [51,52] 

Our database analysis also reveals that the classic AMPs are not the first rich AMPs discovered in nature. By conducting a database search based on the year of publication, we have re-constructed the timeline of discovery for rich AMPs (summarized in Table 5). Gramicidin A, discovered in the 1930s, is identified as the first AMP rich in Trp, Val, and Leu residues [53]. It tuned out that frog PGLa is the first Ala-rich AMP based on our definition [54]. While rabbit α-defensins are the first Arg-rich AMPs [54], human histatins are the first antimicrobial peptides rich in histidine [26]. Additional first members of rich AMPs [55,56,57,58,59,60,61,62,63] can be found in Table 5. 

Our study shines light on the deployment of rich AMPs in different life kingdoms. In bacteria, only Gly-rich, Leu-rich, and Val-rich AMPs have 10 or more members out of 385 bacteria peptides (Table 1). Despite a total of 368 plant peptides, rich AMPs are rare in this kingdom (Table 1). This is likely due to the fact that many plant AMPs possess a folded structure frequently stabilized by multiple disulfide bonds [12,64]. Such folded structures more resemble globular proteins, where a variety of amino acids are required to achieve a proper folding of the polypeptide chain. In animals, rich AMPs are abundant covering nine amino acids, which are Ala, Cys, Gly, His, Ile, Lys, Leu, Pro, and Arg. Of note is that Leu-rich AMPs are much more abundant in animals than either Ile-rich or Val-rich AMPs (Table 1). This observation is in line with the results from our previous in vitro peptide mutation study. A change of all leucine residues to isoleucine in the database-designed peptide DFTamP1 reduces peptide solubility, while a change of leucine to valine causes a loss in antibacterial activity [65]. Likewise, linearized teixobactin is rich in isoleucine but inactive. However, the peptide gains antimicrobial activity when some isoleucine residues in the enhanced sequence are converted to leucine, reinforcing the importance of leucine in natural AMPs for host defense [66].

Since the majority of natural AMPs in the APD (76%) are animal peptides (Figure 1), we have also mapped out the distribution of rich AMPs in different animal classes. It appears that each animal class deploys a different set of rich AMPs (Table 2). Amphibians contain 46 Ala-rich, 43 Gly-rich, and 152 Leu-rich AMPs, respectively. These rich AMPs provide additional insight into our observation that alanine, glycine, and leucine are frequently occurring amino acids in amphibian AMPs [30]. About 50% of the Leu-rich amphibian peptides are temporins [4]. This explains in part why European frogs possess a higher content of leucine on average than those found from other continents [47]. The logo plot for temporins (Figure 3C) reveals the dominant positions for leucine at the C-terminus. Structurally, the main body of the sequence (residues 3–13) forms a two-turn amphipathic helix, which is stabilized by an N-terminal hydrophobic patch. The proline at position 3 is conserved to facilitate the hydrophobic packing between the N-terminal hydrophobic patch and the amphipathic helix [67]. Such an amphipathic structure with more hydrophobic amino acids but few cationic lysine generates an effective bullet against methicillin-resistant *Staphylococcus aureus* (MRSA) by targeting bacterial membranes (Figure 4A) [35,65]. The overall low percentages of both arginine (1.5%) and histidine (1.1%) in 1150 amphibian AMPs explain why Arg-rich AMPs are rare (only one found) and His-rich AMPs have not been found in amphibians (Table 2). In addition, only two temporins are labeled as “Gly-rich”, indicating this type of rich AMPs belongs to other amphibian AMPs. Indeed, 23 out of 43 are nigrocin peptides. The multiple glycines in the amino acid sequences of nigrocin are highly conserved (Figure 3B). In addition, a highly conserved Rana box sequence **C**^15^GLSGL**C**^21^ is located at the C-terminus of the peptide. The disulfide bond of nigrocin-PN is important for antimicrobial activity, since replacing them with a pair of leucines reduced peptide activity, and the deletion of the entire Rana box made the peptide inactive [68]. However, a replacement of the Rana box with phenylalanine enhanced the activity of nigrocin-HL [69]. Nigrocin can also form an amphipathic helical structure (supported by Circular Dichroism) to eliminate bacteria and their biofilms [68,69]. Finally, of the 46 Ala-rich AMPs, 29 are dermaseptins from South American frogs. This fact contributes to the overall higher content of alanine in South American frogs observed previously [47]. These peptides also form amphipathic helices for antimicrobial action against bacteria, viruses, fungi, and parasites [70]. Interestingly, we observed a clear length-dependent amino acid use in these helical dermaseptins [71]. Shorter peptides with 21–25 residues have a slightly lower net positive charge than longer ones (31–35 residues). This results from a decrease in lysine content. In contrast, alanine increases in proportion to peptide length, while leucine decreases.

Insects are closest to amphibians in terms of the deployment of rich AMPs (Table 2). They share Ala-rich, Gly-rich, and Leu-rich AMPs, although they are not identical. In the Leu-rich family, there are eight mastoparans with 14 residues. These helical peptides kill both bacteria and fungi [39]. Different from amphibians, there are more Lys-rich AMPs in insects. Notably, Lys-rich AMPs are also deployed in reptiles, spiders, and chelicerata (>10 AMPs), although they are rare in mammals. However, mammals are the only animals that are abundant in Arg-rich AMPs (38 members), since there are six or less such peptides in other classes. These Arg-rich AMPs are mostly defensins (17 members) and cathelicidins (18 members), which are the two major families of host defense peptides in mammals. It appears that His-rich AMPs constitute another family frequently deployed in mammals (15 counts) but less frequently found in other animals such as fish (five counts in Table 2).

Like insects, mammals also use Pro-rich AMPs as host defense molecules. Two-thirds of mammal Pro-rich AMPs are cathelicidin peptides such as Bac5, Bac7, and PR-39 [29,72]. Some Pro-rich peptides from insects and mammals have been shown to inhibit protein synthesis by binding to bacterial ribosomes [41,42]. The Pro-rich peptides in the extended conformation serve as a plug to block the exit of the newly synthesized polypeptide. These Pro-rich examples indicate a similar molecular design, and the working mechanism can be shared by invertebrates and vertebrates to combat invading pathogens. In addition, a Pro-rich domain may be combined with another rich domain to create a modular design. Of the 22 AMPs with such a design, 13 contain a Pro-rich domain. In crustaceans, the Pro-rich domain is usually located at the N-terminus followed by a Cys-rich domain [73]. It remains to be tested whether the Pro-rich region in multiple domain AMPs works in the same manner by inhibiting protein synthesis.

Mammals also deploy small Cys-rich peptides to control pathogens. In the APD, there are 16 natural θ-defensins, which are smaller than either α-defensins or β-defensins [60]. Theta-defensins present a remarkably unique amino acid signature [74], where the polar group (Thr, Ser, Gln, and Asn, and Tyr, based on the APD) is represented only by threonine, the glycine/proline group (Gly and Pro) uses only glycine, and the charge group (Lys, Arg, His, Asp, and Glu) contains solely arginine. Only the hydrophobic group (Leu, Val, Ile, Ala, Met, Phe, Trp, and Cys) is diversified with five types of amino acids: leucine, isoleucine, valine, alanine, and cysteine in this family of circular peptides. These Cys-rich defensins retain the three disulfide bonds as observed in α and β-defensins. Moreover, the N- and C-termini are connected via a peptide bond, making θ-defensins very stable as a potential therapeutic molecule [60]. Hepcidin peptides from mammals and fish are also Cys-rich AMPs. Different from θ-defensin, the β-sheet structure of hepcidin (Figure 4B) is stabilized by four disulfide bonds. Human hepcidin has only modest antimicrobial activity. It is proposed that hepcidin is the long-sought hormone that regulates and maintains the homeostasis of iron in humans [75]. Our findings here raise new questions for future studies. What is the reason for each animal class to deploy different types of rich AMPs? Why are Arg-rich and His-rich AMPs, but not Lys-rich AMPs, abundant in mammals? Alanine, glycine, leucine, and lysine have been recognized as frequently occurring amino acids in amphibians [30]. Then, what is the reason that only Lys-rich AMPs are sparse in amphibians, while Ala-rich, Gly-rich, and Leu-rich peptides are all abundant (Table 2)?

Our understanding of rich AMPs in nature may also inspire the design of novel antimicrobials. Based on our analysis, we propose the following rules:

Rule 1: Met-, Asn- and Gln-rich AMPs have not been found in nature to date. These amino acids may be avoided in designing rich antimicrobial peptides. Even in “not rich” peptides, a change of methionine to other aliphatic amino acids improves the stability of pediocin PA-1 [48].

Rule 2: Since we found only short Asp-rich and Phe-rich AMPs, it may be useful to avoid the design of longer peptides rich in these amino acids.

Rule 3: The preferred association with arginine can be useful for designing novel Trp-rich, Cys-rich, and Pro-rich peptides simultaneously rich in arginine.

The preferred amino acid association in rich AMPs may also be useful in peptide design. We found 14 AMPs rich in both arginine and proline. In addition, of the 11 Trp-rich AMPs, five are rich in both Trp and Arg. There are 47 Cys-rich AMPs. Only eight AMPs are rich in both Arg and Cys (Figure 3A). They are all θ-defensins. Thus, arginine can be a partner in Pro-rich, Cys-rich, and Trp-rich AMPs. Biologically, arginine can confer antimicrobial activity [76,77,78]. In contrast, none of these rich AMPs are simultaneously rich in lysine or histidine. There are other types of rich amino acid association as well. Leu-rich AMPs can be accompanied by alanine (7 entries), lysine (7 entries), glycine (13 entries), valine (3 entries), and histidine (2 entries). Simultaneous Leu- and Ala-rich AMPs, as well as simultaneous Leu- and Gly-rich AMPs, do occur in amphibians and insects. Gramicidins B and C are both Leu- and Val-rich peptides from bacteria. Remarkably, gramicidin A is rich in three types of amino acids: Trp, Val, and Leu (Table 5). Note that the seven peptides rich in both leucine and lysine are all made in laboratories, indicating a lack of such antimicrobial examples in nature, which is probably due to unwanted toxicity to host cells [79,80].

Reductionists have utilized lysine and leucine as representative basic and hydrophobic amino acids to design amphipathic helical peptides [79,80]. Interestingly, the APD reveals that lysine, leucine, and glycine are frequently occurring amino acids in natural AMPs. These amino acids enabled the design of GLK-19, which is rich in both lysine and leucine [30]. Glycine appears to be an important residue in AMPs, conferring flexibility and selectivity to polypeptide chains. In linear amphibian AMPs, both glycine and lysine increase proportionally with an increase in peptide length, whereas leucine decreases [71]. Adepantin-1, rich in glycine, is a highly selective peptide obtained from computing design [81]. Gellman and colleagues found that the incorporation of a small flexible glycine-like unit into bicomponent polymers improves the cell selectivity of ternary polymers [82]. Practically, synthetic peptides (obtained from library screen or de novo design) [3] designed in this manner can be easier to make, as they require only a few types of amino acids. Although not always appreciated, numerous designer peptides in the literature are actually rich AMPs (Table 6). For instance, combi-1 identified from a library screen is rich in arginine [83]. DFTamP1, a peptide designed based on the database filtering technology, is rich in leucine [65]. While the K4 peptide is rich in both lysine and phenylalanine [84], the antibiofilm peptide GL13K is rich in lysine [85]. The lens-coating peptide, melamine, is rich in arginine [86]. Some of these rich peptides have been demonstrated as being useful to treat wounds as topical agents [36,85].

In addition, several rich AMPs show systemic efficacy in vivo. Pro-rich AMPs, possessing low hydrophobic and high cationic amino acids, have been demonstrated to show in vivo efficacy against Gram-negative pathogens by inhibiting protein synthesis [32,87]. In contrast, database-designed helical peptides, such as DFTamP1, with high hydrophobic and low cationic amino acids, also show systemic efficacy against Gram-positive pathogens such as MRSA in neutropenic mice [35]. While horine, a Trp-rich peptide with a novel amphipathic helical structure, protects mice from MRSA infection, verine, with a spiral structure, can kill both Gram-positive MRSA and the Gram-negative *Klebsiella* pathogen [34]. There are also other designed peptides that are rich in various amino acids (Table 6) [88,89,90,91]. Some rich AMPs from natural sources are also promising. Bacterial teixobactin, rich in isoleucine, has been reported as a novel antibiotic with demonstrated in vivo activity [33]. Cys-rich RTD-1 from monkeys is a small β-sheet defensin with in vivo efficacy as well [37]. Hence, several types of rich AMPs are promising future antibiotics. In fact, there are already rich AMPs in use. Gramicidin A (Table 5) is the first peptide antibiotic in clinical use. Colistin, the last resort antibiotics against Gram-negative pathogens, is a cyclic peptide highly rich in basic 2,4-diaminobutanoic acid (60%), which is a side chain shortened lysine.

## 4. Materials and Methods

### 4.1. The Antimicrobial Peptide Database

Since the APD was online in 2003 [92], this original database has been expanded in numerous aspects, ranging from peptide entries to search functions. The new database features are detailed in versions 2 (APD2) [30] and 3 (APD3) [8] published in 2009 and 2016, respectively. Some more recent additions to the APD have also been described [7,47]. The APD3 is an important version, since it defined a set of criteria for data registration. It also established a systematic classification of antimicrobial peptides into six life kingdoms: bacteria, archaea, protists, fungi, plants, and animals. Thus, the APD3 houses a core data set for natural AMPs, facilitating both research and education in the field [8]. When this study was completed, there were 3425 peptides in the APD3, which is nearly a seven-fold increase in peptide number compared to the first version [92]. This database laid a solid foundation for us to map the landscape of natural AMPs and to understand their design principles [47].

During this analysis, the entire database was re-annotated by associating peptides discovered from the six life kingdoms with a universal search key “natural AMPs” in the NAME field of the APD. These “natural AMPs” were mostly obtained via the classic isolation and characterization methods or through sequence homology search in genomes. These “natural AMPs” did not include natural peptides that have not been tested for antimicrobial activity. This database annotation opens the door to our registration of additional genome-coded peptides predicted by various in silico approaches (e.g., machine learning/artificial intelligence) and validated by antimicrobial assays. Whether such “predicted” peptides are true natural AMPs remains to be validated. As a consequence, these peptides are placed under the same umbrella “predicted” in the APD. The genetic source of the peptide is also indicated (bacteria, microbiota, or human) if the sequence is not altered. If the peptide made differs from the natural sequence, it is also annotated as “synthetic”. In the current APD, there are eight “synthetic” peptides which are variants of the “predicted” sequences.

### 4.2. Definition and Exceptions

We define “rich AMPs” here as having the repetitive appearance of one or more amino acids in the sequence with a percentage greater than 25%. There are exceptions, however. *For peptides with less than 10 amino acids*, 25% was easily reached, leading to an over-prediction. For example, the two different amino acids in the shortest two-residue peptide would occupy 50% for each. Because neither amino acid is actually rich, we further define at least three identical amino acids in order to be “rich” for short peptides with less than 10 amino acids. Another exception is *AMPs with multiple domains*. An amino acid may be rich in a localized segment but not globally, leading to an under-prediction. These AMPs are treated as another special group annotated as “modular design” (searchable in the NAME field). Of the 22 “modular design” AMPs in the APD, 13 were annotated with one or two rich domains as reported in the literature [93,94].

Note that the amino acid contents (e.g., His and Trp) for some classic rich AMPs are less than 25%. Since they have been recognized as such in the literature for years, we decided to keep them in the database as “amino acid-rich classic”. For instance, human histatin 3 contains 22% histidine [26]. In addition, our analysis did not include those peptides with incomplete amino acid sequences (labeled as BWQ in the Name field of the APD). Finally, we did not assign the label “rich” to peptides that are made of nonstandard amino acids.

### 4.3. Identification of Rich AMPs in the APD Database

In order to identify rich AMPs in the APD, we downloaded all the peptide sequences in the FASTA format (3425 as of 3 June 2022) and conducted an amino acid compositional calculation by using the R programming language. The calculations were self-consistent as evidenced by a sum of 100% for the percentages of the 20 standard amino acids. To further validate the results, we also made comparisons with the results calculated in the prediction interface of the APD [8,92]. For a known sequence in the APD, the calculation can be made after subtle sequence shuffling. Our calculations identified all amino acid-rich antimicrobial peptides in an unbiased manner. Based on the “definitions and exceptions” above, all the identified rich AMPs were then annotated in the APD in the format “Xxx-rich”. To obtain a list of any rich AMP, one can enter “Xxx-rich” (e.g., Pro-rich) into the NAME field of the database followed by search. For instance, we found 80 peptides when “Pro-rich” was searched.

### 4.4. Amino Acid Homopolymer Probing in the APD

If AMPs had evolved from amino acid homopolymers, there would be residual examples in nature. To identify natural AMPs most resembling the molecular probe, we conducted sequence probing in the APD. The probes consist of a string of each amino acid (viz, homopolymers such as AAAAA or DDDDD), ranging from 5 to 50 amino acids at a step size of 5. The peptide with the highest similarity was recorded and plotted. The plots depict the similarity of AMPs to peptide probes as a function of the probe length.

### 4.5. Structure and Activity Search in the APD

Both structure and activity information can be searched in the APD since the first version [92]. By “structure”, we mean NMR and X-ray crystallography determined structures. For AMPs, NMR structures dominate in the APD (392 unique NMR structures and 58 crystal structures as of 2022). The helical structure has increased substantially (258 currently) in the APD due to the annotation of those conformations suggested by circular dichrosim (CD). AMPs rich in certain amino acids pose a challenge to NMR structural determination primarily due to the limitation of spectral resolution. For these peptides, it is useful to utilize the improved 2D NMR method [95] that expands proton-only spectra to ^15^N and ^13^C correlated spectra [34,35]. In the APD, we found 61 structures with coordinates for rich AMPs (19%). Although the APD programmed the original structural classification (α, β, and rich) in the prediction interface, we have executed a unified structural classification scheme [96] based on the presence and absence of α and β structure: α, β, αβ, and non-αβ (see the Results for further details). The non-αβ structure is broad and can include any type of structure or random coils not covered by the first three classes. While the original database [92] programmed five types of activity search (i.e., antibacterial, antifungal, antiviral, anticancer, and hemolytic), the activity search function has increased to 26 types recently [7,8], allowing us to better map the activity spectrum for each rich AMP family known to date.

### 4.6. Tools for Identification of Rich Antimicrobial Peptides

The APD programmed a prediction interface in 2003 where each input sequence is predicted to be an α-helical, β-sheet, or “rich” peptide based on Boman’s initial classification [9]. The 25% definition was incorporated into the APD some time ago [7]. Hence, for a new peptide, one can use the APD Calculation and Prediction tool (https://aps.unmc.edu/prediction accessed on 3 June 2022) to determine whether the peptide is rich in any of the 20 standard amino acids. To identify rich AMPs from a large data set with thousands of peptides, users can request the R program from the Wang lab.

## 5. Conclusions

It is now established that the amino acid use in antimicrobial peptides is biased. This is reflected in the amino acid composition of all natural AMPs in the APD with glycine, leucine, and lysine being more abundant (≈10%), while methionine and tryptophan are less frequent (<2%) [6,30]. In the extreme cases, certain amino acids can be dominant so that the sequence is entirely hydrophobic or hydrophilic [53,58]. By defining the word “rich” (amino acid content > 25%), AMPs can be categorized into two classes: “rich” and “not rich”. Our systematic analysis of 3425 peptides in the APD indicates that rich AMPs account for 21%. Ten amino acids (Ala, Cys, Gly, His, Ile, Lys, Leu, Pro, Arg, and Val) are recognized as frequently occurring, seven amino acids (Asp, Glu, Phe, Ser, Thr, Trp, and Tyr) are recognized as occasionally occurring, and three (Met, Asn, and Gln) have not yet been observed. Our analysis has expanded the landscape of rich AMPs from a few classic amino acids to 17 amino acids. Leucine and glycine are the top two most common amino acids in forming rich AMPs (100–200 counts found in the APD). While there are more rich AMPs in bacteria (16%) and animals (24%), they are surprisingly rare in plants (3%). Our study also shines light on the current deployment of rich AMPs in different animal classes. Amphibians and insects share Ala-rich, Gly-rich, and Leu-rich AMPs, but they differ in Lys-rich and Pro-rich peptides. While His-rich and Arg-rich AMPs are common in mammals, Lys-rich AMPs are rare. Although Pro, Cys, and Trp-rich AMPs can also be abundant in arginine, it is peculiar that AMPs rich in both lysine and leucine have not been found in nature based on the APD. A possible reason is the toxicity of the peptide when these two amino acids are simultaneously rich. These findings are useful for designing novel AMPs. Indeed, out of the 11 amino acids utilized in synthesizing artificial rich AMPs found in the APD (i.e., lab designed peptides), nine amino acids are overlapping with those frequently observed in naturally rich AMPs (Table 1). Hence, scientists have already mimicked nature’s wisdom in making rich AMPs. We anticipate new rich AMPs will continue to be discovered in future studies [97,98,99]. Because of the sequence simplicity and demonstrated potency in animal models, we also anticipate that some rich AMPs will move into clinical trials and eventually become novel antibiotics.

## Figures and Tables

**Table 1:** Statistics of amino acid rich antimicrobial peptides in bacteria, plants, and animals.

Peptide	Bacteria	Plants	Animals	Total ^a^	Average Rich% ^b^	Man-Made ^c^
Ala-rich	5	0	75	80	29.0%	4
Arg-rich	6	5	63	74	29.2%	11
Asn-rich	0	0	0	0	NA	0
Asp-rich	0	0	5	5	84.6%	0
Cys-rich	3	0	41	44	23.1%	3
Gln-rich	0	0	0	0	NA	0
Glu-rich	2	0	1	3	28.6%	0
Gly-rich	15	4	85	104	41.1%	2
His-rich	0	1	31	32	25.1%	0
Ile-rich	2	0	15	17	30.9%	1
Leu-rich	11	1	200	212	32.8%	12
Lys-rich	5	0	59	64	32.5%	21
Met-rich	0	0	0	0	NA	0
Phe-rich	1	0	5	6	32.4%	2
Pro-rich	4	2	73	79	29.5%	1
Ser-rich	2	0	6	8	28.2%	0
Thr-rich	3	0	0	3	29.5%	0
Trp-rich	1	0	5	6	28.6%	5
Tyr-rich	1	0	1	2	28.3%	0
Val-rich	11	0	3	14	32.4%	1

^a^ Data were obtained from the APD (https://aps.unmc.edu) on 3 June 2022. The total rich AMPs from bacteria, plants and animals. Those with more than 10 counts are italicized. These amino acids have also been used by humans to design rich AMPs. ^b^ The averaged rich amino acid percentage of the total rich AMPs from natural sources in the database. NA, not available due to a lack of such peptides. Only cysteine gave an average below 25% due to the presence of a Cys-rich domain in multiple domain AMPs. ^c^ Data from the APD based on selected synthetic peptides.

**Table 2:** Deployment of rich antimicrobial peptides in animals ^a^.

Peptides	Vertebrates					Invertebrates				
	Amphibians	Birds	Fish	Mammals	Reptiles	Chelicerata	Crustacean	Insects	Mollusca	Spiders
Ala-rich	46	0	4	3	3	0	0	15	2	0
Arg-rich	1	3	0	38	3	5	2	1	6	1
Cys-rich	1	0	12	19	0	0	9	0	0	0
Gly-rich	43	0	8	1	1	6	5	9	0	5
His-rich	0	0	5	15	0	0	1	2	0	0
Ile-rich	6	0	2	0	0	4	0	2	0	1
Leu-rich	152	0	3	4	0	2	0	35	0	1
Lys-rich	5	0	4	2	15	11	0	13	2	10
Pro-rich	2	0	0	21	1	0	18	28	3	0

^a^ Obtained from the APD (https://aps.unmc.edu).

**Table 3:** Structures of rich antimicrobial peptides ^a^.

Rich AMPs	α-Helix	β-Sheet	αβ	Non-αβ
Ala-rich	20	0	0	0
Arg-rich	9	14	1	2
Asn-rich	0	0	0	0
Asp-rich	0	0	0	0
Cys-rich	1	7	1	0
Gln-rich	0	0	0	0
Glu-rich	0	0	0	0
Gly-rich	7	2	0	1
His-rich	7	0	0	0
Ile-rich	6	1	0	0
Leu-rich	58	1	0	0
Lys-rich	38	2	0	0
Met-rich	0	0	0	0
Phe-rich	2	0	0	0
Pro-rich	0	0	0	2
Ser-rich	0	0	0	0
Thr-rich	0	0	0	0
Trp-rich	3	0	0	3
Tyr-rich	0	0	0	0
Val-rich	4	0	0	0

^a^ Obtained from the APD (https://aps.unmc.edu).

**Table 4:** Biological activities of rich antimicrobial peptides ^a^.

Peptides	Antibacterial			Antifungal	Antiviral	Antiparasitic
	Gram-Positive (G+)	Gram-Negative (G−)	Both (G+/−)			
Ala-rich	6	7	70	25	3	8
Arg-rich	6	11	81	39	11	1
Asn-rich	0	0	0	0	0	0
Asp-rich	0	2	5	0	0	0
Cys-rich	11	2	39	30	11	1
Gln-rich	0	0	0	0	0	0
Glu-rich	1	0	2	1	1	0
Gly-rich	10	22	83	46	7	0
His-rich	0	3	22	21	3	0
Ile-rich	5	1	16	7	2	0
Leu-rich	70	8	200	77	7	10
Lys-rich	4	6	80	30	3	5
Met-rich	0	0	0	0	0	0
Phe-rich	2	1	8	2	0	0
Pro-rich	12	19	75	22	0	4
Ser-rich	3	3	8	1	0	0
Thr-rich	3	0	3	0	1	1
Trp-rich	0	2	11	3	2	0
Tyr-rich	1	0	2	2	0	0
Val-rich	3	0	15	1	2	0

^a^ Obtained from the APD (https://aps.unmc.edu). Examples for each type of antimicrobial activity of each rich peptide can be found in the APD.

**Table 5:** The discovery timeline of the first amino acid rich antimicrobial peptides ^a^.

APD ID	Name	Amino Acid Sequence	Rich ^b^	Year	Ref
499	Gramicidin A	**V**GALA**VVV***W*L*W*L*W*L*W*	27% *Trp*, 27% **Val**, 27% Leu	1939	[53]
187	Defensin NP-1	VVCAC*RR*ALCLP*R*E*RR*AGFC*R*I*R*G*R*IHPLCC*RR*	30% *Arg*	1983	[55]
1227	Microcin B17	V*G*I*GGGGGGGGGG*SC*GG*Q*GGG*C*GG*CSN*G*CS *GG*N*GG*S*GG*S*G*SHI	60% *Gly*	1986	[25]
798	Histatin 1	DS*H*EKR*HH*GYRRKF*H*EK*HH*S*H*REFPFYGDY GSNYLYDN	18% *His*	1986	[26]
210	PGLa	GM*A*SK*A*G*A*I*A*GKI*A*KV*A*LK*A*L	33% *Ala*	1988	[54]
9	Cathelicidin Bac 5	RFR*PP*IRR*PP*IR*PP*FY*PP*FR*PP*IR*PP*IF*PP*IR*PP*F R*PP*LG*P*F*P*	47% *Pro*	1989	[23]
2143	Pln149	YSLQMGATAI*K*QV*KK*LF*KKK*GG	27% *Lys*	1994	[56]
321	Uperin 2.7	G*II*D*I*AKKLVGG*I*RNVLG*I*	26% *Ile*	1996	[57]
528	SAAP fraction 3	*DDDDDDD*	100% *Asp*	1996	[58]
812	Enkelytin	FA*E*PLPS*EEE*G*E*SYSK*E*PP*E*M*E*KRYGGFM	28% *Glu*	1996	[59]
445	RTD-1	GF*C*R*C*L*C*RRGV*C*R*C*I*C*TR	33% *Cys*	1999	[60]
357	Japonicin-1	*FF*PIGV*F*CKI*F*KTC	29% *Phe*	2002	[61]
1491	NLP-31	QW**G***Y***GG***Y***G**R**G***Y***GG***Y***GG***Y***G**R**G***Y***GG***Y***GG***Y***G**R **G***Y***GG***Y***G**R**G**M*Y***GG***Y***G**RP*Y***GG***Y***G**W**G**K	26% *Tyr*, 53% **Gly**	2004	[62]
1647	PZN	RC**T**C**TT**II*SSSS***T**F	29% *Ser*, 29% **Thr**	2011	[63]

^a^ Obtained from the APD (https://aps.unmc.edu). ^b^ Amino acid fonts correspond to those in the sequence.

**Table 6:** Eleven amino acids used in making rich antimicrobial peptides in laboratories.

Peptide	Amino Acid Sequence	Rich Amino Acids	Ref.
Combi-1	RRWWRF	Arg	[83]
Adepantin-1	GIGKHVGKALKGLKGLLKGLGES	Gly	[81]
GLK-19	GLKKLLGKLLKKLGKLLLK	Leu, Lys	[30]
DFTamP1	GLLSLLSLLGKLL	Leu	[65]
K4 peptide	KKKKPLFGLFFGLF	Lys, Phe	[84]
GL13K	GKIIKLKASLKLL	Leu, Lys	[85]
Api137 ^a^	Gu-ONNRPVYIPRPRPPHPRL	Pro	[87]
Horine	WWWLRRRW	Trp, Arg	[34]
Verine	RRRWWWWV	Trp, Arg	[34]
WLBU2	RRWVRRVRRWVRRVVRVVRRWVRR	Val, Arg	[88]
Ovispirin	KNLRRITRKIIHIIKKYGPTILRIIRIIG	Ile	[89]
DP1	KLAKLAKKLAKLAK	Ala, Lys, Leu	[90]
Retrocyclin-1	GICRCICGRGICRCICGR	Cys	[91]

^a^ gu = N,N,N’,N’-tetramethylguanidino and O is ornithine.

## Data Availability

Relevant data for this analysis can be obtained from the antimicrobial peptide database (http://aps.unmc.edu/AP accessed on 3 June 2022).
